# Contractile apparatus in CNS capillary pericytes

**DOI:** 10.1117/1.NPh.9.2.021904

**Published:** 2022-01-24

**Authors:** Şefik E. Erdener, Gülce Küreli, Turgay Dalkara

**Affiliations:** Hacettepe University, Institute of Neurological Sciences and Psychiatry, Ankara, Turkey

**Keywords:** pericyte, actin, contractility, central nervous system, capillary, blood flow

## Abstract

**Significance:**

Whether or not capillary pericytes contribute to blood flow regulation in the brain and retina has long been debated. This was partly caused by failure of detecting the contractile protein α-smooth muscle actin (α-SMA) in capillary pericytes.

**Aim:**

The aim of this review is to summarize recent developments in detecting α-SMA and contractility in capillary pericytes and the relevant literature on the biology of actin filaments.

**Results:**

Evidence suggests that for visualization of the small amounts of α-SMA in downstream mid-capillary pericytes, actin depolymerization must be prevented during tissue processing. Actin filaments turnover is mainly based on de/re-polymerization rather than transcription of the monomeric form, hence, small amounts of α-SMA mRNA may evade detection by transcriptomic studies. Similarly, transgenic mice expressing fluorescent reporters under the α-SMA promoter may yield low fluorescence due to limited transcriptional activity in mid-capillary pericytes. Recent studies show that pericytes including mid-capillary ones express several actin isoforms and myosin heavy chain type 11, the partner of α-SMA in mediating contraction. Emerging evidence also suggests that actin polymerization in pericytes may have a role in regulating the tone of downstream capillaries.

**Conclusions:**

With guidance of actin biology, innovative labeling and imaging techniques can reveal the molecular machinery of contraction in pericytes.

## 1 Introduction

Since their first identification by Rouget,^1^ pericytes have been hypothesized to have a role in capillary blood flow regulation. This view was challenged by several counter arguments over the decades.^2^^–^^5^ Now, it is generally agreed that blood flow reaching to the capillary network is regulated at the precapillary sphincter and first-order capillary by contraction/relaxation of the sphincter and pericytes ensheathing the first-order capillary.^6^^–^^11^ Several lines of evidence have disclosed that pericytes are contractile *in vivo* such as the upstream vascular smooth muscle cells (vSMCs) as originally suggested.^6^^,^^7^^,^^11^^–^^13^ Pericytes located over the first- to fourth-order capillaries and their junctions, all of which exhibit high luminal coverage and α-smooth muscle actin (α-SMA) expression, have convinced most researchers that they might regulate the blood flow by contracting or relaxing.^6^^–^^11^^,^^14^ On the contrary, there is skepticism that the mid-capillary pericytes on downstream (≥ fourth order) capillaries, which show the morphological characteristics of mesh or thin-strand pericytes,^15^ could play a contractile role and contribute to blood flow regulation because their thin processes provide only limited mural coverage and express little or no α-SMA.^4^^,^^7^^,^^16^ Some technical limitations currently preclude unambiguous clarification of these points as reviewed below.

First, capillary radius changes induced by downstream pericytes are heterogeneous. As reported by Hall et al.,^8^ while responders to sensory stimulation may dilate by 14% in the whisker cortex, two-thirds of the downstream capillaries either do not dilate or the diameter change induced is likely near the limit of optical resolution under *in vivo* imaging conditions. For a 5-μm capillary (most downstream capillaries are smaller than 5  μm), a 10% change in luminal diameter results in a 500-nm difference, which can correspond to a few pixels under commonly used two-photon microscopy acquisition conditions. Motion artifacts caused by brain pulsations, respiration or movement of unanesthetized animals further complicate the specificity of measurements. Indeed, following the pioneering study by Hall et al.,^8^ two later studies, while replicating the rapid and robust response of the upstream capillaries to whisker stimulation, concluded that the downstream pericytes were not significantly responsive to sensory (relaxation) or optogenetic (contraction) stimulation.^4^^,^^17^ Nevertheless, using advanced optics with higher magnification and resolution as well as analysis tools, more recent studies succeeded to detect subtle luminal diameter changes in high-order capillaries *in vivo*, which typically exhibited dilation or constriction with slow onset but with prolonged duration in response to physiological (a few seconds of odor exposure)^10^ and optogenetic stimulation,^6^ respectively. Although smaller dilations (<5%) induced by physiological stimulation in the olfactory bulb or retina was difficult to assess compared to more robust changes induced by 1-min optogenetic stimulation in whisker cortex (by 20%), corresponding alterations in intra-pericytic calcium or capillary blood flow were suggestive that luminal changes measured were not artifactual.^10^^,^^12^ These minor diameter changes in downstream capillaries can indeed appreciably facilitate the increase in blood flow because most of the vascular bed resistance originates from small capillaries.^18^ The dilations observed are likely to be passive secondary to upstream hydrostatic effects but may also be eased by active, sluggish relaxation of downstream pericytes.^10^ Supporting the presence of a contractile capacity in downstream pericytes, a small and slowly developing luminal narrowing induced by a vasoconstrictive agent was demonstrated under more stable in vitro conditions in whole mount retina.^7^ Similarly, electrical stimulation of the fifth-order capillaries off the radial retinal arterioles (taken as zero order) in whole-mount retina caused a contraction by 12% along with an increase in pericyte calcium, which rapidly propagated to upstream pericytes via gap junctions.^19^ A propagating constriction upon stimulation of capillary pericytes including the downstream ones near the inner plexiform layer in whole-mount retina had also been reported by Peppiatt et al.^20^ Rapid signal transmission from downstream pericytes, which have an ideal position to monitor neuronal activity, to up and downstream endothelial and mural cells via gap junctions^19^ or intercellular tunnels^12^ can orchestrate timing of capillary diameter changes along the arteriolovenular axis to provide an optimal blood flow for the active synapses nearby downstream capillaries.^10^ Therefore, simultaneous temporal profiling of the luminal changes together with pericyte calcium transients could be highly informative to better understand the neurovascular coupling dynamics in the cortex *in vivo* as well, but currently remains technically challenging.

The second problem is difficulty in detecting small amounts of α-SMA or its mRNA in downstream pericytes. α-SMA, the contractile actin isoform in vSMCs is drastically reduced in pericytes located on capillaries downstream to the first three to four branch orders both in the brain and retina when the first microvessel branching off the cortical penetrating or radial retinal arterioles is numbered as the first branch order capillary.^7^^,^^16^ The absence of internal elastic lamina and presence of protruding ovoid cell bodies (bump-on-a-log appearance) seems to be the best criteria to define the first capillary harboring a pericyte and may help aligning the branch order numbers in different tissues.^7^^,^^15^^,^^21^ As discussed above, subtle but active tonus modifications in downstream capillaries may be needed to ensure homogenization of the flow rate between neighboring branches during functional hyperemia, although they seem to be passively dilating.^6^^,^^22^ Moreover, restoration of the basal tonus after passive distension might also require α-SMA-mediated active contraction. Supporting such possibilities, Shih’s group has recently demonstrated that high-order pericytes did contract when optogenetically stimulated (contractile capability) and that their ablation led to dilation of the underlying capillary (contribution to tonus).^6^ Strongly suggesting that these observations were not caused by experimental laser injury, ablation of a proximal pericyte bridging to a downstream distant pericyte led to dilation in the downstream capillary unaffected by the laser beam.^6^ The contractility to downstream mid-capillary pericytes can be provided by small amounts of α-SMA, whose presence has been disclosed using rapid tissue fixation techniques or toxins that inhibit actin depolymerization and/or promote actin polymerization in the retina.^23^^–^^25^ Suggesting a physiological role for actin de/re-polymerization in downstream pericytes, intravitreal administration of vasoconstrictive agent noradrenalin *in vivo* induced actin polymerization in these pericytes.^25^^,^^26^ The actin stabilizing approaches also unveiled that 60% of NG2-DsRed-positive pericytes expressed α-SMA [six times more than detected with paraformaldehyde (PFA) fixation] in the intermediate layer of the retina, whose capillaries (corresponding to the seventh to eighth orders) show the greatest dilation during light stimulation, whereas pre-venullary capillaries remained α-SMA-negative.^23^^,^^27^ Very recently, Nelson’s group has revealed that all pericytes along the arteriolovenular axis expressed Myh11, the motor protein partner of α-SMA in realizing actomyosin-mediated contraction, although they could not detect α-SMA in downstream pericytes in whole-mount retinas fixed with the slowly acting fixative PFA.^7^ The small pool of α-SMA constantly de/re-polymerizing in downstream pericytes is likely maintained by a slow actin monomer turnover, as is usually the case for actin in other cells.^28^ The latter view is consistent with low α-SMA mRNA reported^29^^,^^30^ and also with low levels of fluorescent protein/reporter expression under α-SMA promoter in downstream pericytes.^4^^,^^7^ Transgenic mouse lines expressing membrane-bound mCherry driven by the α-SMA promoter have been shown to faithfully recapitulate endogenous α-SMA expression rate in various embryo and adult mouse tissues.^31^ Indeed, not only mCherry but also GCaMP expression under the control of Acta2 promoter is reportedly low in downstream pericytes,^7^ supporting the idea of a weak α-SMA promoter activity rather than rapid degradation of α-SMA mRNA or suppression of translation by regulatory mechanisms. In other words, because the small pool of α-SMA protein in downstream pericytes is mainly replenished by de/repolymerization rather than breakdown/synthesis of α-SMA monomers, transcriptional and translational activity for α-SMA is expectedly to be low. Regulation of α-SMA expression involves a complex interaction of multiple positive and negative regulatory elements that act in a cell-type specific manner such that the precise combinatorial interactions of *cis* and *trans* acting factors determine the outcome of expression in different cells and under various physiological conditions.^32^^–^^34^ For example, myocardin, a potent promoter of Acta2, is expressed 134 times less in pericytes compared to arteriolar smooth muscle cells.^29^^,^^30^ Supporting a low mRNA content, RNA knockdown with RNA interference in the retina preferentially affected α-SMA expression in pericytes downstream to the fourth-order.^23^ As noted above, immunohistochemistry performed after prevention of actin depolymerization shows that not all downstream pericytes are α-SMA-positive in the retina,^23^^,^^25^ which might have diluted the average hit numbers of α-SMA-mRNA from mid-capillary pericytes of the brain.^35^ As mentioned above, this heterogeneity in contractile capability of downstream pericytes was also noted in their response to sensory stimulation in the cortex^8^ and cautions against treating this population of pericytes as a homogenous group (e.g., in diameter or mRNA statistics).

In summary, before concluding that downstream pericytes do not express α-SMA and are not contractile, further research seems to be warranted. While this research solves the technical challenges working with such small vessels and cells, it should also take the basic actin physiology, well characterized in cell biology, into account. Hence, we will summarize some basics of actin physiology below, which we hope may contribute to the experimental designs aiming at studying downstream pericytes.

## 2 Actin Isoforms in Pericytes

Actin, an evolutionarily well-conserved microfilament, is the most abundant protein (20% of the total cellular protein) in mammalian cells except for a few types.^28^^,^^36^^,^^37^ As a structural protein, its turnover is very slow, counted by weeks in muscle cells.^28^ Only 7% of the filamentous actin (F-actin) is formed from newly synthesized monomers, otherwise, the primary source is polymerization of the existing pool of monomeric globular actin (G-actin).^38^ G-actin has six mammalian isoforms, which are encoded by different genes but share a highly similar amino acid sequence (95% homology): the contractile α-skeletal muscle actin, α-cardiac muscle actin, α-SMA, γ-enteric SMA isoforms and the cytoskeletal, γ-cytoplasmic actin, and β-cytoplasmic actin isoforms. Most of the difference stems from the amino acid sequence at the N-terminal region, which determines the affinity to actin binding proteins that maintain the population of assembly ready actin monomers (e.g., profilin), regulate polymerization (e.g., ADF/cofilin, profilin), block the growing ends of actin filaments (e.g., gelsolin), form a nucleus for actin assembly (e.g., gelsolin, Arp2/3, and cofilin), sever actin filaments (e.g., gelsolin, ADF/cofilin), and cross-link actin filaments (e.g., Arp2/3).^36^^,^^39^^,^^40^

**Fig. 1:**
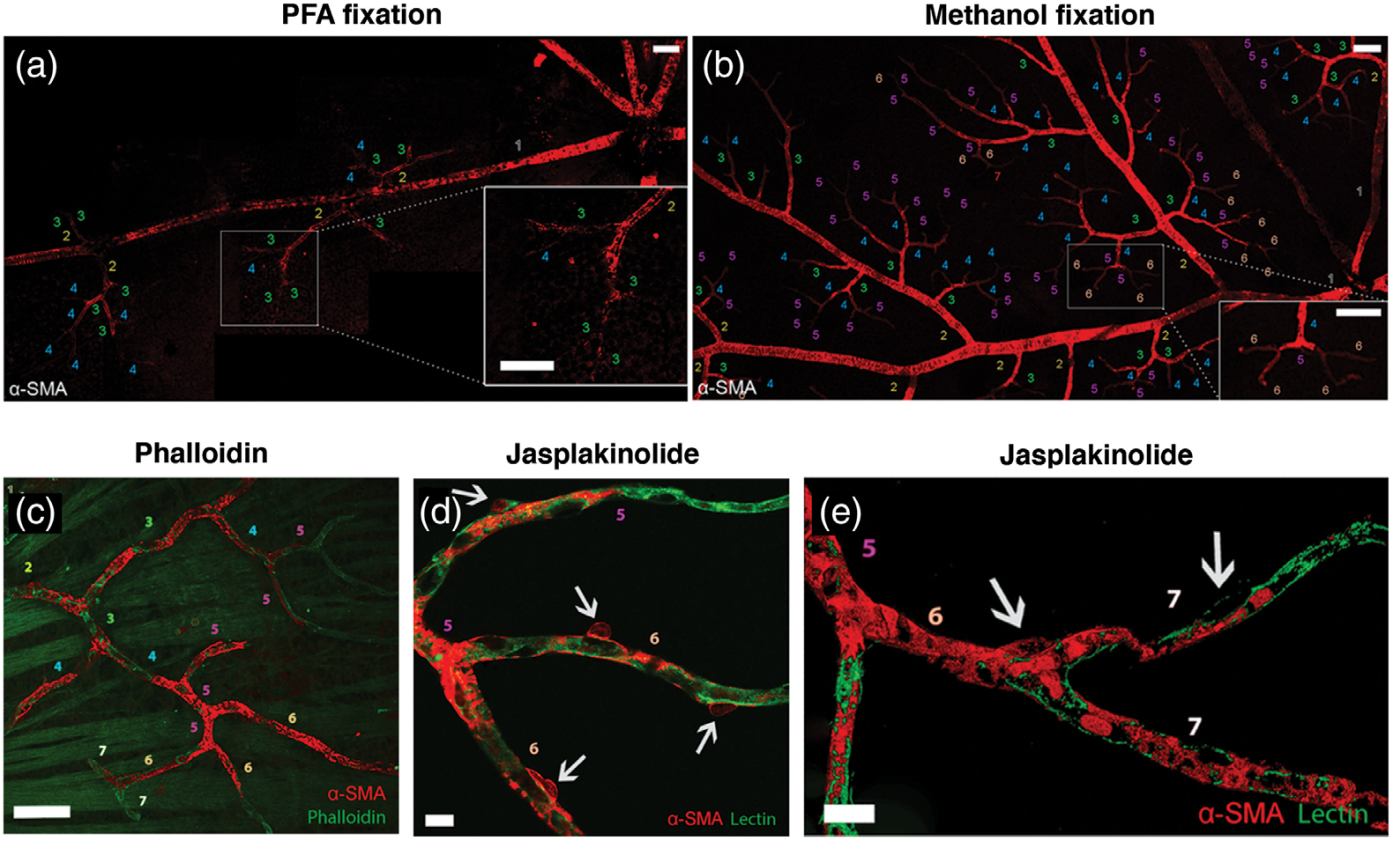
Comparison of α-SMA immunolabeling in retinal capillary pericytes under (a) PFA and (b) −20°C methanol fixation. Each capillary segment is numbered corresponding to its branching order. Faster fixation of retinas with methanol at −20°C shows clear α-SMA immunoreactivity in up to sixth order microvessels. Prevention of α-SMA depolymerization *in vivo* with phalloidin or jasplakinolide revealed further α-SMA immunolabeling in high order retinal capillaries. (c) Phalloidin, intravitreally injected for preventing F-actin depolymerization *in vivo*, was fluorescent-tagged (green) and revealed α-SMA immunolabeling (red) in sixth and seventh order retinal capillaries on whole-mount retinas *ex vivo*. (d) and (e) F-actin stabilization *in vivo* with Jasplakinolide further increased the number of α-SMA-positive pericytes and an enhanced α-SMA immunolabeling intensity by promoting polymerization as well as inhibiting depolymerization (red) in sixth and seventh order retinal capillaries, which were visualized with lectin (green). It is noteworthy that the branch ordering in this study took the central retinal artery but not the radial retinal arterioles as the zero-order (also in other retina figures below), hence, the branch numbers are one order higher compared to the studies on cerebral vasculature, which should be considered when comparing results. After jasplakinolide, not all downstream pericytes were α-SMA-positive, but the ratio of α-SMA-positivity within NG2-DsRed-positive pericytes increased to 78.7%, 60.4%, and 55.0% in the superficial, intermediate and deeper retinal vascular layers, respectively. Arrows point to pericyte somas and numbers indicate the branch order. Image in (e) is surface rendered. Scale bars: 40  μm in (a)–(c), 10  μm in (d) and (e). Adapted from Alarcon-Martinez et al.^23^ with permission.

Cytoplasmic and smooth muscle isoforms of actin were detected in cultured pericytes.^41^ Although mid-capillary pericytes were previously reported not to express α-SMA in tissue sections or *in vivo*,^3^^,^^4^ the presence of α-SMA in high-order pericytes has been disclosed after prevention of F-actin depolymerization,^23^^,^^25^ suggesting that α-SMA filaments are actively de/re-polymerized in pericytes and their rapid depolymerization during tissue processing before fixation hampers detection of thin, dispersed α-SMA fibers by immunolabeling unlike the thick bundles of α-SMA in upstream pericytes^23^^,^^24^^,^^42^^,^^43^ (Fig. 1). The latter studies^23^^,^^24^ also showed that short interfering RNA (α-SMA-siRNA) suppressed α-SMA expression preferentially in high branch order (downstream) capillary pericytes and prevented their contraction, conforming to the existence of a smaller pool of α-SMA involved in contraction of downstream capillary pericytes. These findings were confirmed by recent single-cell RNA sequence of the cells of brain vasculature, which found a large amount of α-SMA mRNA in vSMCs, whereas low hits were detected in pericytes.^30^^,^^44^

## 3 Visualizing Actin in Fixed and Live Tissues

**Fig. 2:**
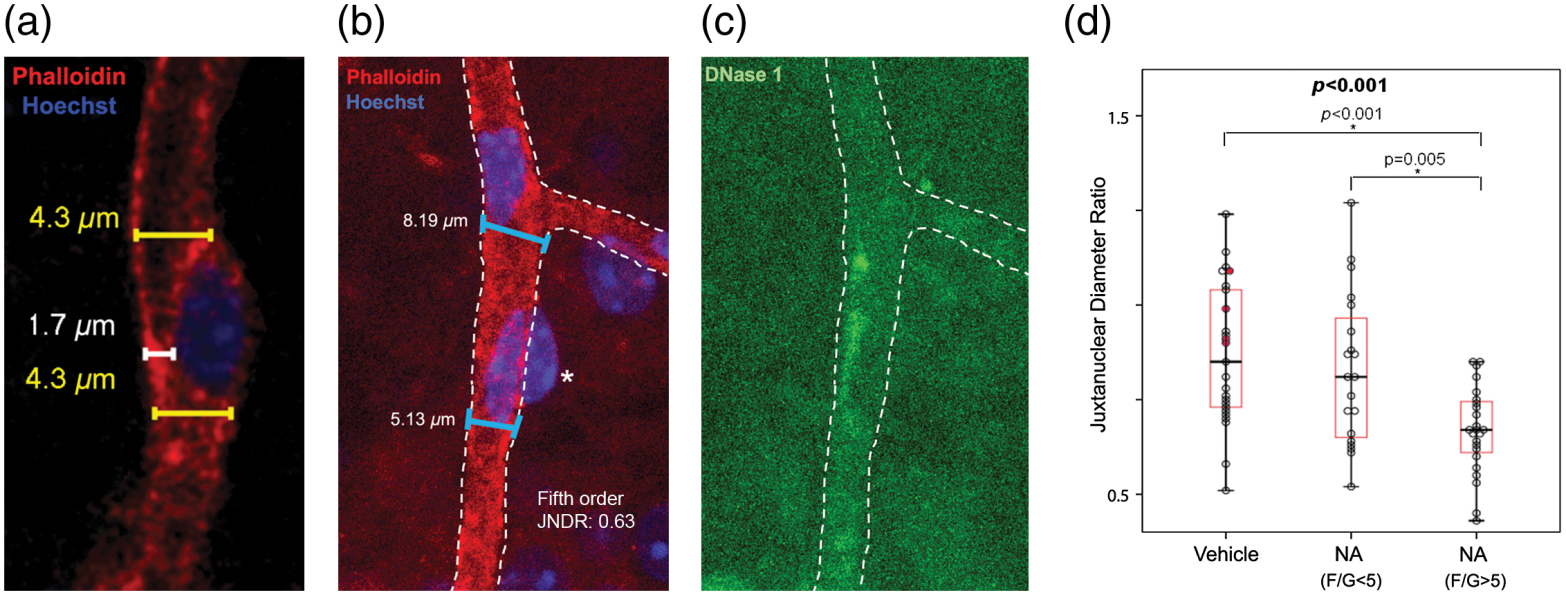
Noradrenalin-induced actin polymerization is associated with capillary diameter decrease. Filamentous actin (F-actin) was labeled with phalloidin and globular actin (G-actin) with DNase I. (a) Image illustrates how juxtanuclear capillary diameter was measured. Measurements under the soma (white) were not preferred because they give misleading values in some instances where the pericyte nucleus is superimposed on vessel lumen in maximum projection images. Juxtanuclear values were then divided by the initial diameter of each capillary segment at the proximal branching point to yield the juxtanuclear diameter ratio. (b) and (c) The ratio of the diameter near pericyte soma to the diameter at the branch origin is defined as the juxtanuclear diameter ratio. Image illustrates how these diameters are measured in a fifth-order pericyte with F/G-actin signal ratio above 5. Asterisk in (b) shows the index pericyte’s soma. JNDR: juxtanuclear diameter ratio. (d) In vascular orders of 5 and 6, which had significantly increased F/G-actin signal ratio values in response to noradrenaline administered into the vitreous, a cutoff F/G value of 5 was able to differentiate noradrenaline and vehicle treated groups. See Kureli et al.^25^ for details. Figure and part of its legend were reproduced from Kureli et al.^25^ with permission.

Actin is found in polymerized [polymeric/filamentous(F)] or depolymerized [monomeric/globular(G)] forms. Phalloidin, obtained from the mushroom *Amanita phalloides*, binds to monomers in actin filaments by interacting with two neighboring monomers.^45^^,^^46^ Therefore, fluorescently conjugated form of phalloidin makes F-actin visible but not G-actin [Figs. 1(c), 2(a), 2(b), and 4(a)]. Affinity of phalloidin is unaffected by the length of actin filaments or the interaction with tropomyosin and myosin because of its small molecular size (0.6 nm),^45^^–^^47^ ensuring reproducible labeling independent of the physiological state. However, it should be noted that phalloidin cannot differentiate between actin isoforms; for the latter purpose, specific immunofluorescent staining is currently the only solution. SiR-actin is a recently introduced cell-permeable alternative^48^ that can help live tissue imaging, but it has F-actin stabilization effect, hence, can interfere with actin dynamics in live cells. Whereas phalloidin binds to filamentous actin, deoxyribonuclease I (DNase I) enzyme has a natural affinity for the monomeric form.^49^ It binds to G-actin with 1:1 ratio and its enzymatic activity stops upon binding. Its fluorescently conjugated forms are used in combination with phalloidin to assess monomeric and polymeric states of actin.

Immunofluorescent staining with antibodies against actin is the conventional method of labeling actin in fixed tissues.^50^ Although nonspecific binding and background staining could sometimes be problematic, this is the only method for differentiating actin isoforms, such as α-SMA and β-actin. Background labeling in the cytoplasm partly originates from antibodies binding to freely dispersed G-actin in addition to the filamentous structures. The major challenge for immunofluorescent labeling of α-SMA filaments is their tendency to rapidly depolymerize before tissue fixation when using slow fixatives such as PFA as mentioned above. Low amounts of loosely organized α-SMA filaments in mid-capillary pericytes appear to be particularly vulnerable compared to the α-SMA filaments in upstream pericytes and vSMCs.^23^ Whether or not this vulnerability has a biological significance is currently unknown. However, these pericytes show substantial F-actin polymerization^25^ and slowly contract when stimulated compared to upstream pericytes,^6^ suggesting that F-actin de/re-polymerization may have a particular role in mid-capillary pericytes. The labeling performance of antibodies is dependent on the fixation method;^51^ for instance, methanol fixation is superior for labeling α-SMA in retinal tissues^23^ and a combination of formaldehyde, glutaraldehyde and saponin is superior for staining β-actin in rat adenocarcinoma cells.^52^ It should be noted that methanol fixation might not be applicable for the brain tissue due to its high lipid content. The comparison of fixatives for imaging pericyte α-SMA in different tissues, including the brain, requires further research.

A different set of tools is required for actin imaging in live cells and tissues. GFP tagging of monomeric actin was developed but GFP-actin was not efficiently incorporated into the polymerized form.^53^ Therefore, genetically encoded actin-binding peptides derived from organisms such as yeasts or humans were fused to fluorescent proteins. LifeAct, a 17-aminoacid peptide from yeast fused with GFP, is such a tool that labels actin in eukaryotic cells.^54^ LifeAct can be expressed in cultured cells and a transgenic mouse line that expresses LifeAct fused to eGFP is also available.^55^ LifeAct has been successful to label F-actin in axon terminals and growth cones,^56^ in endothelial cells of retina and also in vSMCs and pericytes of skin and skeletal muscle.^57^ Recently, LifeAct has been reported to interfere with actin dynamics *in vivo*, impacting stress fiber functioning and cytoskeletal architecture.^58^^,^^59^ Some actin binding proteins, like cofilin, have also been shown to interfere with binding of LifeAct to F-actin.^60^ Because LifeAct, binds to both F-actin and G-actin,^51^^,^^54^ unlike phalloidin, background fluorescence may be a problem.

Alternative probes are also available. Utrophin (UtrCH), an actin binding protein from humans, can bind to actin globules, hence; serve as a genetically encoded label for F-actin.^61^ It has not yet been tested in nervous system, but in *Drosophila*, a concentration-dependent disintegration of actin cytoskeleton has been reported.^62^ Experience with another probe, F-tractin from the rat,^63^ is relatively limited compared to the others. But its size is considerably larger than LifeAct and UtrCH, hence, can interfere with other F-actin binding proteins and alter actin cytoskeleton structure.^51^^,^^64^ It should be noted that not all strategies label the same actin subsets in live cells. For instance, LifeAct cannot be incorporated into filopodia-like structures, whereas GFP-actin can be. On the other hand, LifeAct can better demonstrate stress fibers than GFP-actin.

## 4 Actin Polymerization and Depolymerization

There is a highly dynamic balance between two actin forms, F-actin and G-actin in live cells, which rapidly shifts toward the polymerized form to promote contractility upon mechanical or chemical stimulation.^65^^,^^66^ Steady-state polymerization-depolymerization activity, that is, adding new monomers to the barbed end while removing monomers from the pointed end of filaments, is called “treadmilling” and the growth rate is generally slower than 1 subunit per second.^28^^,^^67^ This mechanism provides motility to cells by way of formation of lamellipodia. The durability of filaments may range from seconds to days depending on the activity; for example, having a faster turnover in lamellipodia and more stable state in myofibrils. Polymerization rate is also affected by local monomer concentrations. The concentrations are heterogenous in polarized cells, and local translation events contribute to this heterogeneity.^68^

F-actin polymerization is one of the universal cellular mechanisms providing contractility, motility, and tonus; therefore, it likely plays a role in pericyte contraction as well, as does it in vSMCs^25^^,^^69^ (Fig. 2). Unlike upstream pericytes, rapid depolymerization of α-SMA suggests that polymerization may regulate the length of contractile α-SMA filaments in downstream pericytes.^25^ Polymerization of cytoskeletal actins can also function as an independent but parallel mechanism to actomyosin-mediated contraction by providing cellular stiffness against mechanical deformation of vSMCs during contraction and a lattice between contracting stress fibers and extracellular matrix to transform the tension developed by actomyosin cross bridge cycling to vessel constriction.^65^^,^^70^^–^^72^ It likely contributes to vSMC tonus because blockage of actin polymerization induces relaxation in vessels without affecting actomyosin-mediated contraction.^73^ Rho kinase, in addition to the actomyosin-mediated contraction, also regulates actin polymerization via activation of LIM kinase, which phosphorylates cofilin, preventing depolymerization.^71^^,^^74^ This pathway has recently been shown to be involved in optogenetically-induced slow contraction of downstream pericytes and suggested to play a role in pericyte tone as well.^6^ Supporting this view, another activator of the Rho kinase pathway, vasoconstrictor noradrenalin has also been shown to induce actin polymerization in downstream pericytes.^25^ The slow and sustained contraction and relaxation characteristics of downstream pericytes favor a mechanism involving actin de/re-polymerization especially in the prolonged phase as well as in pericyte tonus. However, further research is needed to elucidate the relative contribution and timing of actomyosin- and polymerization-mediated contraction/relaxation mechanisms in pericytes.

Differential labeling of F-actin and G-actin in the same cell (see below) allows calculation of F/G actin ratio from fluorescence intensities and this ratio is significantly higher in capillaries with smaller juxtanuclear diameters relative to the segment origin^25^ (Fig. 2).

## 5 Organization of Actin in vSMCs and Pericytes

**Fig. 3:**
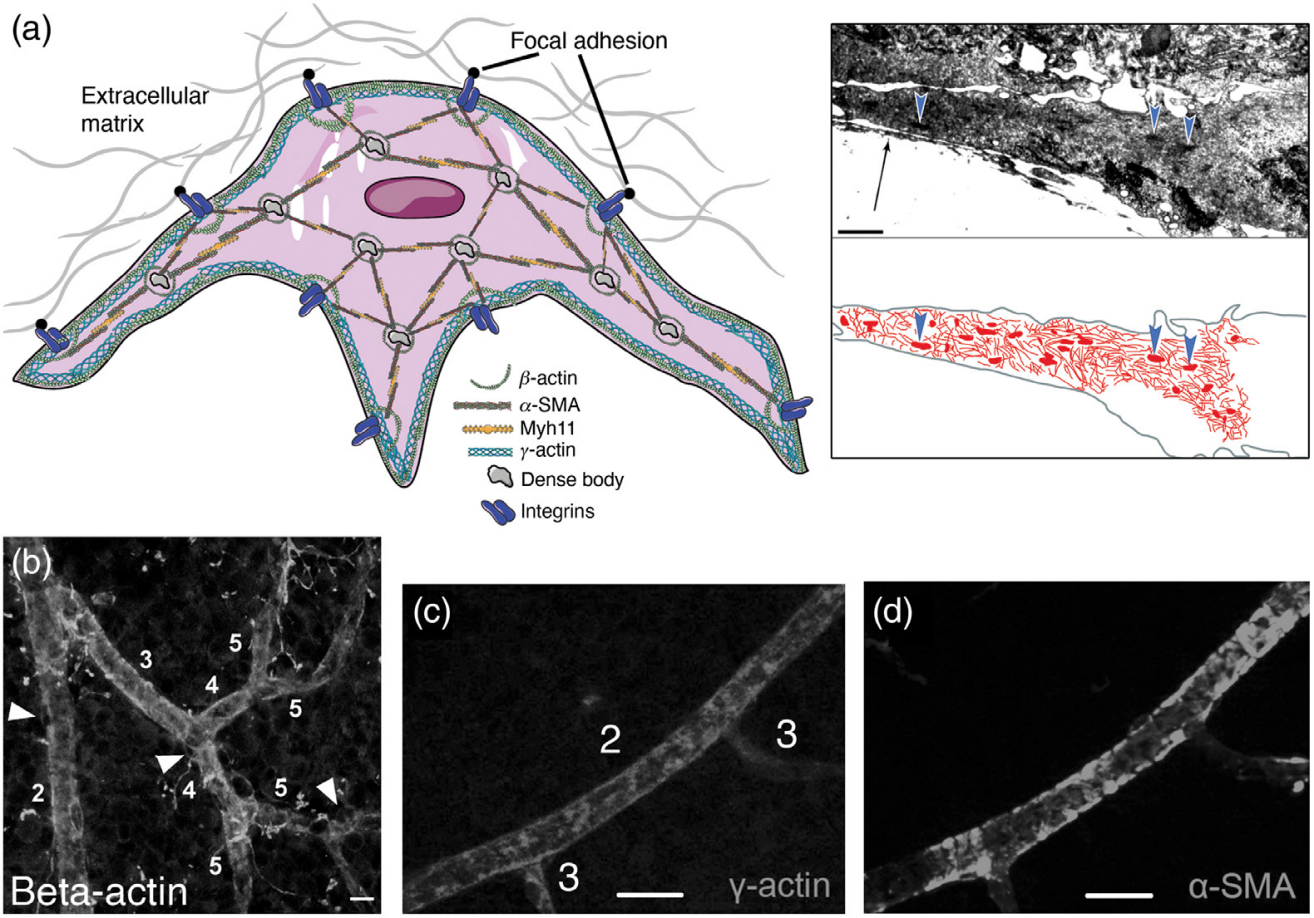
Putative organization of actin filaments in pericytes. The diagram is based on the available pericyte data and is inspired by extensive data from SMCs. β- and γ-cytoplasmic actins are concentrated in cortical/submembranous regions, whereas β-cytoplasmic actin is also located around dense bodies. α-SMA, along with myosin filaments, stretches between focal adhesion points and dense bodies, forming the contractile stress fibers. This figure is produced using Servier medical art (http://www.servier.com). Inset shows a transmission electron micrograph of a cultured retinal pericyte process, harboring actin filaments and dense bodies in cytoplasm (arrowheads). Arrow indicates the extracellular matrix elements. Microfilaments and dense bodies are hand-outlined in red to depict their organization inside the pericyte process. Inset adapted with modifications from Schor et al.^78^ (b) Immunolabeling for cytoskeletal β-actin, which runs along the cortex of the vessel in addition to diffuse labeling. Scale bar: 10  μm. Adapted from Kureli et al.^25^ (c) and (d) γ- cytoplasmic actin and α-SMA distribution in vessels after intravitreally injected phalloidin. Despite phalloidin stabilization of F-actin filaments, gamma-actin remained detectable only on ≤fourth-order branches unlike α-SMA. Scale bar: 20  μm. (c) and (d) Adapted from Alarcon-Martinez et al.,^23^ with permission.

We can take vSMCs as the proxy cells to gain insight into the contractile machinery and actin cytoskeleton in pericytes. In vSMCs, actin filaments are attached to focal adhesion points (dense plaques) on the plasma membrane to transmit the intracellular forces to the extracellular matrix^69^^,^^75^ (Fig. 3). The connection to extracellular matrix generates the constrictive force necessary to narrow the vessel lumen.^72^^,^^76^ Phosphorylation of focal adhesion associated proteins (e.g., vinculin, paxillin, and talin) upon mechanical stimulation starts a reaction that results in actin polymerization.^69^^,^^77^ The intracellular anchoring points of F-actin bundles are called dense bodies, which also contain α-actinin for cross-linking the filaments.

**Fig. 4:**
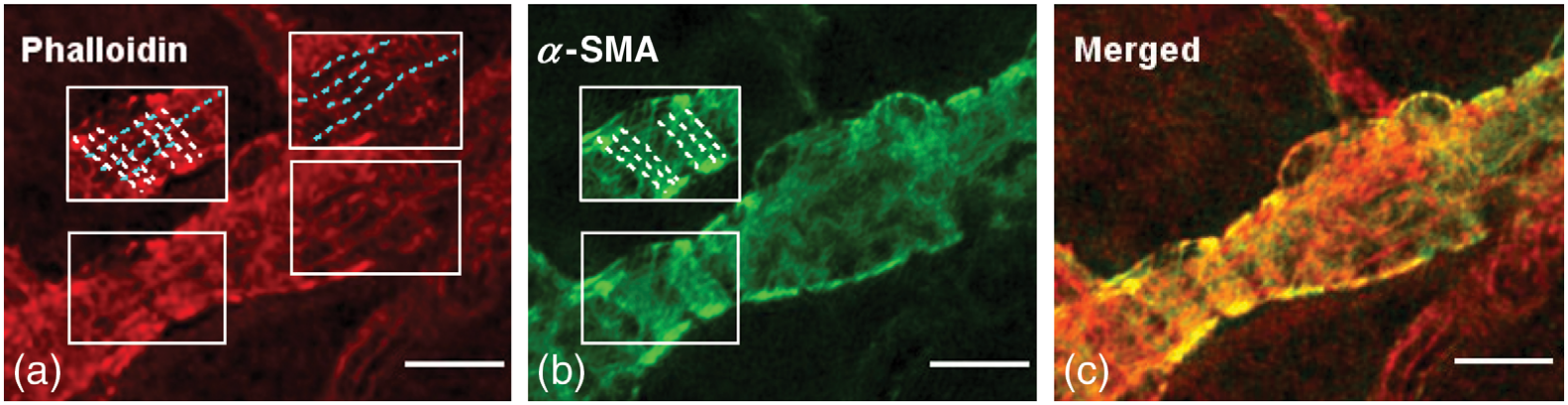
(a)–(c) α-SMA in the processes of a pericyte on the third-order retinal vessel runs around the vascular axis (white-dashed lines), whereas non-α-SMA isoforms of actin can be traced along the longitudinal axis of the vessel (cyan-dashed lines). It is noteworthy that the absence of α-SMA immunostaining of the phalloidin-positive longitudinal filaments in contrast to circular ones in inset. Scale bar: 10  μm. Adapted from Kureli et al.,^25^ with permission.

**Fig. 5:**
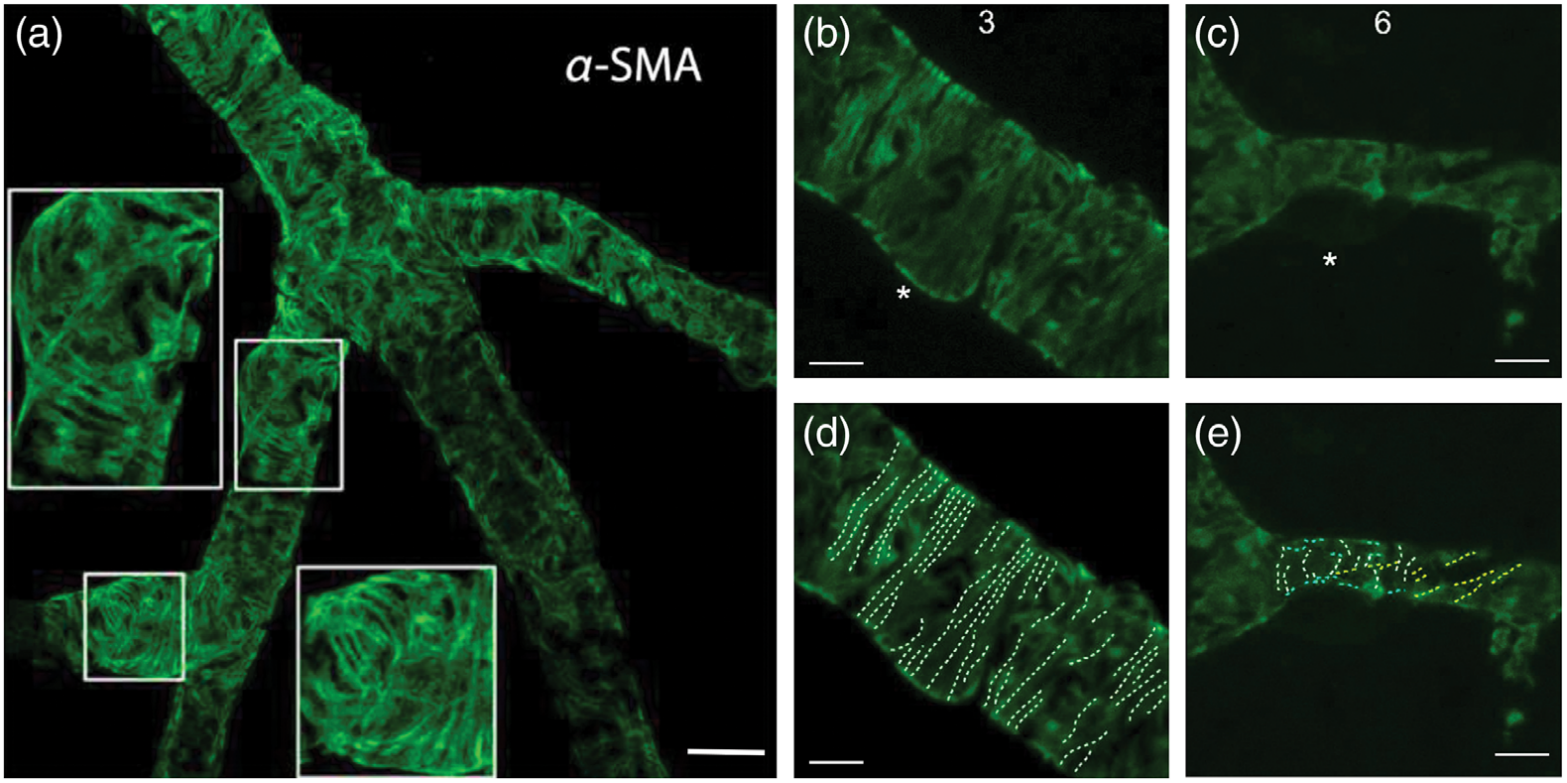
(a) α-SMA fiber bundles form circular string-like structures reminiscent of contractile stress fiber organization. Insets: 2× magnified. (b)–(e) Bundles are differentially organized in pericytes from third and sixth retinal vascular orders. The organization of the α-SMA labeling within the cytoplasm of pericyte processes suggest a vectoral structure, functioning in capillary diameter changes. It is noteworthy that in the (b) and (d) third order, bundles regularly run rather circumferential (white), while in the (c) and (e) sixth-order bundle orientation becomes irregular with some bundles running oblique (yellow) or parallel (cyan) to the longitudinal capillary axis. Scale bar: 10  μm in (a), 5  μm in (b-e).

In vSMCs, contractile and cytoskeletal actin filaments are differentially organized.^69^ α-SMA together with myosin II forms the contractile structures called stress fibers.^72^ Stress fibers in vSMCs are less orderly compared to sarcomeres of skeletal muscles such that stress fibers spreading out in different directions are anchored to cell membrane via focal adhesion points as well as to dense bodies in the cytoplasm^69^^,^^72^^,^^79^ (Fig. 3). Stress fibers formed by α-SMA are also reported in pericytes; however, their organization is not as extensively studied as in vSMCs^80^ (Figs. 4 and 5). Pericytes do not express the contractile γ-SMA isoform mRNA (Actg2), which is the predominant form of the stress fibers in enteric and urethral smooth muscle cells.^44^ Of the cytoskeletal isoforms, β-actin stretches along the cortex under the plasma membrane and is concentrated around dense bodies in the cytoplasm, whereas γ-cytoplasmic actin is mainly localized to the cortical (submembranous) region of the cell^36^^,^^69^^,^^72^ (Fig. 3). β and γ cytoplasmic actins constitute more dynamic actin populations compared to α-SMA.^36^ Cytoskeletal actin isoforms contribute to contractility by reorganizing the cytoskeleton for better force transmission, as shown in isolated large cerebral arteries.^37^^,^^72^^,^^81^^,^^82^ A parallel organization of cytoskeletal actin isoforms to the vSMCs has been detected in cultured bovine retinal microvascular pericytes in vitro^41^^,^^80^^,^^83^ and in whole mount retinas *ex vivo*^23^^,^^25^ (Figs. 3 and 4). Transmission electron microscopy of rat and human brain pericytes also disclosed the presence of actin filament network organized around focal adhesion points and dense bodies in the plasma membrane and cytoplasm, respectively.^84^^,^^85^

## 6 Pharmacological Tools for Modulating Actin Dynamics

A number of pharmacological agents, mostly natural toxins, are commonly used to investigate actin polymerization-depolymerization dynamics (Table 1). Besides their role in modulating actin-related cellular mechanisms, these can also serve as tools to improve their microscopic imaging. Phalloidin, which binds to filamentous actin as noted above, stabilizes its structure by inhibiting its depolymerization.^45^^,^^46^ Pretreatment with fluorescent-tagged phalloidin before perfusing the animal improves F-actin labeling in pericytes by preventing depolymerization and help detect F-actin without immunostaining.^23^ Sea sponge-derived jasplakinolide, which stabilizes F-actin and additionally induces polymerization can also further improve F-actin detection in mid-capillary pericytes expressing small/undetectable amounts of α-SMA^23^^,^^86^ (Fig. 1).

**Table 1:** Pharmacological agents for manipulation of actin polymerization.

Natural toxin	Action	Net effect
Latrunculin	Binds G-actin monomers to prevent polymerization	Depolymerization
Cytochalasin	Binds to barbed (+) end of F-actin to prevent polymerization	Depolymerization
Phalloidin	Stabilizes F-actin by inhibiting depolymerization	Stabilization/polymerization
Jasplakinolide	Stabilizes F-actin and induces polymerization	Polymerization

Other pharmacological tools with different mechanisms are also available to modulate actin machinery. Latrunculins are derived from sea sponge.^87^ They bind to G-actin monomers at a 1:1 ratio and prevent their polymerization.^88^ Cytochalasins, which are derived from fungi, preclude polymerization by binding to the barbed (+) end of actin filaments and preventing addition of new subunits.^89^^,^^90^ The aforementioned drugs have been employed in several studies to induce or suppress actin polymerization.^23^^,^^36^

## 7 Myosin II and Actomyosin Cross-Bridge Cycling

**Fig. 6:**
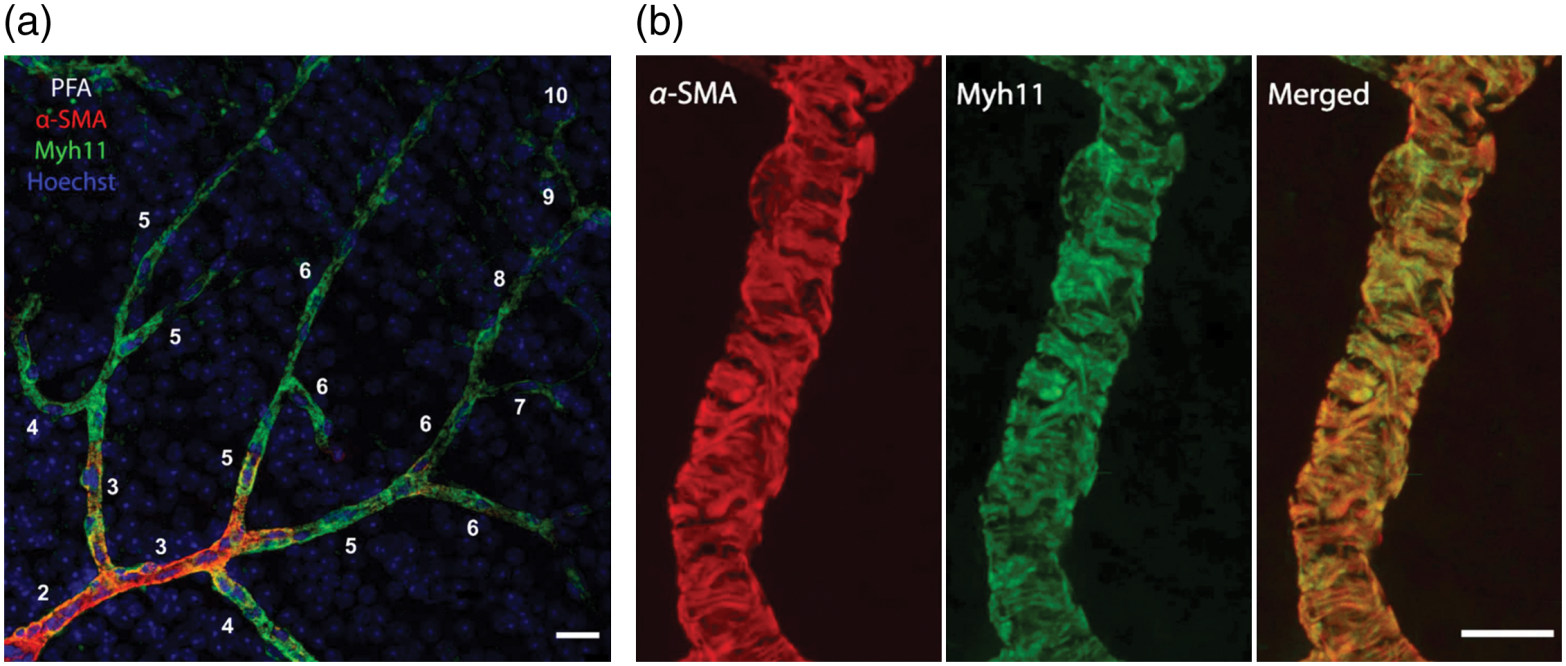
(a) Myh11 labeling is unaffected by fixation method. In this PFA-fixed retina tissue, despite gradual tapering in α-SMA signal toward downstream branches, Myh11 labeling continued all along the whole vascular tree scale bar: 20  μm. (b) The tight overlap between α-SMA and Myh11 proteins is illustrated on a third branch order retinal vessel display the organization of fibrillary bundles in pericyte processes suggestive of tension generating stress fibers. Scale bar: 10-μm. Z-stack maximum projection images of 13-μm thick vascular sections. Use of nanosecondaries for immunolabeling α-SMA and Myh11 provided better resolution and delineation of the fibers by standard confocal microscopy.

Myosin is the principal cellular motor protein. Among 35 subclasses,^91^ myosin II functions in cellular contraction and motility.^92^^,^^93^ Head region of its heavy chain, which is highly conserved across species,^94^ harbors the actin-binding site and ATP catalytic activity required for actomyosin-mediated contraction.^92^ Among the myosin heavy chain isoforms, type 11 is considered to be smooth muscle specific. Pericytes also express myosin II,^95^ and recent studies indicate that the predominant isoform is Myh11^7^^,^^30^^,^^44^^,^^96^ (Fig. 6). Since α-SMA and Myh11 are functionally coupled and tightly co-localized, detecting Myh11 may be particularly useful to study stress fibers in pericytes because myosin does not have the above-mentioned labeling issues for α-SMA and is a stable protein that does not have rapid polymerization-depolymerization dynamics.

Actin-myosin cross-bridge cycling is accepted as the main contractile mechanism for muscle cells. Upon actin-myosin interaction, power stroke moves myosin along the actin filament.^97^ ATP binding to myosin head is essential for detachment of myosin head from actin filament. When bound ATP is hydrolyzed, the cycle restarts and myosin head can bind actin once again. Phosphorylation of regulatory light chain (RLC) of myosin II by myosin light chain kinase, which is activated by cytosolic calcium elevation, is the major regulatory mechanism of contraction in vSMCs.^92^^,^^98^ RLC phosphorylation is also enhanced by inhibition of myosin light chain phosphatase, which is activated by small G protein RhoA/Rho kinase pathway.^99^ Rho kinase also promotes actin polymerization via activation of LIM kinase.^74^ Rho activation induces pericyte contractility in cultures.^71^ Suppression of pericyte contractility with blebbistatin,^96^ a myosin II inhibitor or by fasudil,^6^ a Rho kinase inhibitor suggests that pathways mediating contraction in pericytes are similar to vSMCs.

## 8 Conclusion

Presence of contractile proteins like α-SMA and Myh11, suggests that pericytes on capillaries including the mid-capillary ones may play differentiated roles in blood flow regulation and neurovascular coupling. Whereas the upstream ensheathing type pericytes contribute to regulation of the blood flow to match the tissue demand by contracting and relaxing, the downstream pericytes may facilitate homogenization of capillary flow to optimize the oxygen extraction by subtly changing capillary resistance. Increasing evidence indicates that molecular machinery of the contractile apparatus in pericytes share striking similarities with vSMCs. Application of cutting-edge imaging and labeling techniques together with the well-established knowledge about actin biology in vSMCs, can advance our understanding of neurovascular coupling in microcirculation.

## References

[1] Rouget C., “Memoire sur le developpement la structure et les proprietes physiologiques des capillaires sanguins et lymphatiques,” Arch. Physiol. Norm. Path. 5, 603–663 (1873).

[2] Sims D. E., “The pericyte--a review,” Tissue Cell 18(2), 153–174 (1986). 10.1016/0040-8166(86)90026-1 3085281

[r3] Nehls V. Drenckhahn D., “Heterogeneity of microvascular pericytes for smooth muscle type alpha-actin,” J. Cell Biol. 113(1), 147–154 (1991). JCLBA3 0021-9525 10.1083/jcb.113.1.147 2007619 PMC2288926

[r4] Hill R. A. et al., “Regional blood flow in the normal and ischemic brain is controlled by arteriolar smooth muscle cell contractility and not by capillary pericytes,” Neuron 87(1), 95–110 (2015). NERNET 0896-6273 10.1016/j.neuron.2015.06.001 26119027 PMC4487786

[5] Grutzendler J. Nedergaard M., “Cellular control of brain capillary blood flow: *in vivo* imaging veritas,” Trends Neurosci. 42(8), 528–536 (2019). TNSCDR 0166-2236 10.1016/j.tins.2019.05.009 31255380 PMC7386067

[6] Hartmann D. A. et al., “Brain capillary pericytes exert a substantial but slow influence on blood flow,” Nat. Neurosci. 24(5), 633–645 (2021). NANEFN 1097-6256 10.1038/s41593-020-00793-2 33603231 PMC8102366

[r7] Gonzales A. L. et al., “Contractile pericytes determine the direction of blood flow at capillary junctions,” Proc. Natl. Acad. Sci. U. S. A. 117(43), 27022–27033 (2020). 10.1073/pnas.1922755117 33051294 PMC7604512

[r8] Hall C. N. et al., “Capillary pericytes regulate cerebral blood flow in health and disease,” Nature 508(7494), 55–60 (2014). 10.1038/nature13165 24670647 PMC3976267

[r9] Biesecker K. R. et al., “Glial cell calcium signaling mediates capillary regulation of blood flow in the retina,” J. Neurosci. 36(36), 9435–9445 (2016). JNRSDS 0270-6474 10.1523/JNEUROSCI.1782-16.2016 27605617 PMC5013190

[r10] Rungta R. L. et al., “Vascular compartmentalization of functional hyperemia from the synapse to the pia,” Neuron 99(2), 362–375.e4 (2018). NERNET 0896-6273 10.1016/j.neuron.2018.06.012 29937277 PMC6069674

[11] Grubb S. Lauritzen M. Aalkjaer C., “Brain capillary pericytes and neurovascular coupling,” Comp. Biochem. Physiol. A Mol. Integr. Physiol. 254, 110893 (2021). 10.1016/j.cbpa.2020.110893 33418051

[r12] Alarcon-Martinez L. et al., “Interpericyte tunnelling nanotubes regulate neurovascular coupling,” Nature 585(7823), 91–95 (2020). 10.1038/s41586-020-2589-x 32788726

[13] Alarcon-Martinez L. Yemisci M. Dalkara T., “Pericyte morphology and function,” Histol. Histopathol. 36(6), 633–643 (2021). HIHIES 0213-3911 10.14670/HH-18-314 33595091

[14] Yemisci M. et al., “Pericyte contraction induced by oxidative-nitrative stress impairs capillary reflow despite successful opening of an occluded cerebral artery,” Nat. Med. 15(9), 1031–1037 (2009).1078-8956 10.1038/nm.2022 19718040

[15] Grant R. I. et al., “Organizational hierarchy and structural diversity of microvascular pericytes in adult mouse cortex,” J. Cereb. Blood Flow Metab. 39(3), 411–425 (2019). 10.1177/0271678X17732229 28933255 PMC6399730

[16] Hartmann D. A. et al., “Pericyte structure and distribution in the cerebral cortex revealed by high-resolution imaging of transgenic mice,” Neurophotonics 2(4), 041402 (2015). 10.1117/1.NPh.2.4.041402 26158016 PMC4478963

[17] Cai C. et al., “Stimulation-induced increases in cerebral blood flow and local capillary vasoconstriction depend on conducted vascular responses,” Proc. Natl. Acad. Sci. U. S. A. 115(25), E5796–E5804 (2018). 10.1073/pnas.1707702115 29866853 PMC6016812

[18] Blinder P. et al., “The cortical angiome: an interconnected vascular network with noncolumnar patterns of blood flow,” Nat. Neurosci. 16(7), 889–897 (2013). NANEFN 1097-6256 10.1038/nn.3426 23749145 PMC4141079

[19] Kovacs-Oller T. et al., “The pericyte connectome: spatial precision of neurovascular coupling is driven by selective connectivity maps of pericytes and endothelial cells and is disrupted in diabetes,” Cell Discov. 6, 39 (2020). 10.1038/s41421-020-0180-0 32566247 PMC7296038

[20] Peppiatt C. M. et al., “Bidirectional control of CNS capillary diameter by pericytes,” Nature 443(7112), 700–704 (2006). 10.1038/nature05193 17036005 PMC1761848

[21] Attwell D. et al., “What is a pericyte?” J. Cereb. Blood Flow Metab. 36(2), 451–455 (2016). 10.1177/0271678X15610340 26661200 PMC4759679

[22] Li B. et al., “More homogeneous capillary flow and oxygenation in deeper cortical layers correlate with increased oxygen extraction,” Elife 8, e42299 (2019). 10.7554/eLife.42299 31305237 PMC6636997

[23] Alarcon-Martinez L. et al., “Capillary pericytes express alpha-smooth muscle actin, which requires prevention of filamentous-actin depolymerization for detection,” Elife 7, e34861 (2018). 10.7554/eLife.34861 29561727 PMC5862523

[r24] Alarcon-Martinez L. et al., “Retinal ischemia induces alpha-SMA-mediated capillary pericyte contraction coincident with perivascular glycogen depletion,” Acta Neuropathol. Commun. 7(1), 134 (2019). 10.1186/s40478-019-0761-z 31429795 PMC6701129

[25] Kureli G. et al., “F-actin polymerization contributes to pericyte contractility in retinal capillaries,” Exp. Neurol. 332, 113392 (2020). EXNEAC 0014-4886 10.1016/j.expneurol.2020.113392 32610106

[26] Hirata H. Tatsumi H. Sokabe M., “Mechanical forces facilitate actin polymerization at focal adhesions in a zyxin-dependent manner,” J. Cell Sci. 121(17), 2795–2804 (2008). JNCSAI 0021-9533 10.1242/jcs.030320 18682496

[27] Kornfield T. E. Newman E. A., “Regulation of blood flow in the retinal trilaminar vascular network,” J. Neurosci. 34(34), 11504–11513 (2014). JNRSDS 0270-6474 10.1523/JNEUROSCI.1971-14.2014 25143628 PMC4138352

[28] Pollard T. D., “Actin and actin-binding proteins,” Cold Spring Harb. Perspect. Biol. 8(8) (2016).1943-0264 10.1101/cshperspect.a018226 PMC4968159 26988969

[29] He L. et al., “Analysis of the brain mural cell transcriptome,” Sci. Rep. 6, 35108 (2016). SRCEC3 2045-2322 10.1038/srep35108 27725773 PMC5057134

[30] Vanlandewijck M. et al., “A molecular atlas of cell types and zonation in the brain vasculature,” Nature 554(7693), 475–480 (2018). 10.1038/nature25739 29443965

[31] Armstrong J. J. et al., “Characterization of bacterial artificial chromosome transgenic mice expressing mCherry fluorescent protein substituted for the murine smooth muscle alpha-actin gene,” Genesis 48(7), 457–463 (2010). 10.1002/dvg.20638 20506352 PMC2906650

[32] Mack C. P. Owens G. K., “Regulation of smooth muscle alpha-actin expression *in vivo* is dependent on CArG elements within the 5’ and first intron promoter regions,” Circ. Res. 84(7), 852–861 (1999). CIRUAL 0009-7330 10.1161/01.RES.84.7.852 10205154

[r33] Gan Q. et al., “Smooth muscle cells and myofibroblasts use distinct transcriptional mechanisms for smooth muscle alpha-actin expression,” Circ. Res. 101(9), 883–892 (2007). CIRUAL 0009-7330 10.1161/CIRCRESAHA.107.154831 17823374

[34] Gomez D. Swiatlowska P. Owens G. K., “Epigenetic control of smooth muscle cell identity and lineage memory,” Arterioscler. Thromb. Vasc. Biol. 35(12), 2508–2516 (2015). ATVBFA 1079-5642 10.1161/ATVBAHA.115.305044 26449751 PMC4662608

[35] Chasseigneaux S. et al., “Isolation and differential transcriptome of vascular smooth muscle cells and mid-capillary pericytes from the rat brain,” Sci. Rep. 8(1), 12272 (2018). SRCEC3 2045-2322 10.1038/s41598-018-30739-5 30116021 PMC6095852

[36] Kim H. R. et al., “Cytoskeletal remodeling in differentiated vascular smooth muscle is actin isoform dependent and stimulus dependent,” Am. J. Physiol. Cell Physiol. 295(3), C768–C778 (2008).1522-1563 10.1152/ajpcell.00174.2008 18596213 PMC2544444

[37] Lee S. H. Dominguez R., “Regulation of actin cytoskeleton dynamics in cells,” Mol. Cells 29(4), 311–325 (2010). 10.1007/s10059-010-0053-8 20446344 PMC3910092

[38] Condeelis J. Singer R. H., “How and why does beta-actin mRNA target?” Biol. Cell 97(1), 97–110 (2005). BCELDF 0248-4900 10.1042/BC20040063 15601261

[39] Dugina V. B. Shagieva G. S. Kopnin P. B., “Biological role of actin isoforms in mammalian cells,” Biochemistry (Mosc) 84(6), 583–592 (2019). 10.1134/S0006297919060014 31238858

[40] dos Remedios C. G. et al., “Actin binding proteins: regulation of cytoskeletal microfilaments,” Physiol. Rev. 83(2), 433–473 (2003). PHREA7 0031-9333 10.1152/physrev.00026.2002 12663865

[41] Hoock T. C. Newcomb P. M. Herman I. M., “β actin and its mRNA are localized at the plasma membrane and the regions of moving cytoplasm during the cellular response to injury,” J. Cell Biol. 112(4), 653–664 (1991). JCLBA3 0021-9525 10.1083/jcb.112.4.653 1993736 PMC2288855

[42] Skalli O. et al., “Alpha-smooth muscle actin, a differentiation marker of smooth muscle cells, is present in microfilamentous bundles of pericytes,” J. Histochem. Cytochem. 37(3), 315–321 (1989). JHCYAS 0022-1554 10.1177/37.3.2918221 2918221

[43] Bandopadhyay R. et al., “Contractile proteins in pericytes at the blood-brain and blood-retinal barriers,” J. Neurocytol. 30(1), 35–44 (2001). JNCYA2 0300-4864 10.1023/A:1011965307612 11577244

[44] Zeisel A. et al., “Molecular architecture of the mouse nervous system,” Cell 174(4), 999–1014 e1022 (2018). 10.1016/j.cell.2018.06.021 30096314 PMC6086934

[45] Dancker P. et al., “Interaction of actin with phalloidin: polymerization and stabilization of F-actin,” Biochim. Biophys. Acta 400(2), 407–414 (1975). BBACAQ 0006-3002 10.1016/0005-2795(75)90196-8 126084

[r46] Oda T. Namba K. Maeda Y., “Position and orientation of phalloidin in F-actin determined by X-ray fiber diffraction analysis,” Biophys. J. 88(4), 2727–2736 (2005). BIOJAU 0006-3495 10.1529/biophysj.104.047753 15653738 PMC1305368

[47] De La Cruz E. M. Pollard T. D., “Transient kinetic analysis of rhodamine phalloidin binding to actin filaments,” Biochemistry 33(48), 14387–14392 (1994). 10.1021/bi00252a003 7981198

[48] D’Este E. et al., “STED nanoscopy reveals the ubiquity of subcortical cytoskeleton periodicity in living neurons,” Cell Rep. 10(8), 1246–1251 (2015). 10.1016/j.celrep.2015.02.007 25732815

[49] Hitchcock S. E., “Actin deoxyroboncuclease I interaction. Depolymerization and nucleotide exchange,” J. Biol. Chem. 255(12), 5668–5673 (1980). JBCHA3 0021-9258 10.1016/S0021-9258(19)70681-4 6247341

[50] Lazarides E. Weber K., “Actin antibody: the specific visualization of actin filaments in non-muscle cells,” Proc. Natl. Acad. Sci. U. S. A. 71(6), 2268–2272 (1974). 10.1073/pnas.71.6.2268 4210210 PMC388433

[51] Melak M. Plessner M. Grosse R., “Actin visualization at a glance,” J. Cell Sci. 130(3), 525–530 (2017). JNCSAI 0021-9533 10.1242/jcs.189068 28082420

[52] DesMarais V. et al., “Optimizing leading edge F-actin labeling using multiple actin probes, fixation methods and imaging modalities,” Biotechniques 66(3), 113–119 (2019). BTNQDO 0736-6205 10.2144/btn-2018-0112 30869550

[53] Doyle T. Botstein D., “Movement of yeast cortical actin cytoskeleton visualized *in vivo*,” Proc. Natl. Acad. Sci. U. S. A. 93(9), 3886–3891 (1996). 10.1073/pnas.93.9.3886 8632984 PMC39454

[54] Riedl J. et al., “Lifeact: a versatile marker to visualize F-actin,” Nat. Methods 5(7), 605–607 (2008).1548-7091 10.1038/nmeth.1220 18536722 PMC2814344

[55] Riedl J. et al., “Lifeact mice for studying F-actin dynamics,” Nat. Methods 7(3), 168–169 (2010).1548-7091 10.1038/nmeth0310-168 20195247

[56] Ladt K. Ganguly A. Roy S., “Axonal actin in action: imaging actin dynamics in neurons,” Methods Cell Biol. 131, 91–106 (2016). MCBLAG 0091-679X 10.1016/bs.mcb.2015.07.003 26794509 PMC7336870

[57] Fraccaroli A. et al., “Visualization of endothelial actin cytoskeleton in the mouse retina,” PLoS One 7(10), e47488 (2012). POLNCL 1932-6203 10.1371/journal.pone.0047488 23115648 PMC3480364

[58] Flores L. R. et al., “Lifeact-GFP alters F-actin organization, cellular morphology and biophysical behavior,” Sci. Rep. 9(1), 3241 (2019). SRCEC3 2045-2322 10.1038/s41598-019-40092-w 30824802 PMC6397297

[59] Belyy A. et al., “Structure of the Lifeact-F-actin complex,” PLoS Biol. 18(11), e3000925 (2020). 10.1371/journal.pbio.3000925 33216759 PMC7717565

[60] Munsie L. N. et al., “Lifeact cannot visualize some forms of stress-induced twisted F-actin,” Nat. Methods 6(5), 317 (2009).1548-7091 10.1038/nmeth0509-317 19404250

[61] Burkel B. M. von Dassow G. Bement W. M., “Versatile fluorescent probes for actin filaments based on the actin-binding domain of utrophin,” Cell Motil. Cytoskeleton 64(11), 822–832 (2007). CMCYEO 0886-1544 10.1002/cm.20226 17685442 PMC4364136

[62] Spracklen A. J. et al., “The pros and cons of common actin labeling tools for visualizing actin dynamics during Drosophila oogenesis,” Dev. Biol. 393(2), 209–226 (2014). DEBIAO 0012-1606 10.1016/j.ydbio.2014.06.022 24995797 PMC4438707

[63] Schell M. J. Erneux C. Irvine R. F., “Inositol 1,4,5-trisphosphate 3-kinase A associates with F-actin and dendritic spines via its N terminus,” J. Biol. Chem. 276(40), 37537–37546 (2001). JBCHA3 0021-9258 10.1074/jbc.M104101200 11468283

[64] Belin B. J. Goins L. M. Mullins R. D., “Comparative analysis of tools for live cell imaging of actin network architecture,” Bioarchitecture 4(6), 189–202 (2014). 10.1080/19490992.2014.1047714 26317264 PMC4914014

[65] Gunst S. J. Zhang W., “Actin cytoskeletal dynamics in smooth muscle: a new paradigm for the regulation of smooth muscle contraction,” Am. J. Physiol. Cell Physiol. 295(3), C576–C587 (2008).1522-1563 10.1152/ajpcell.00253.2008 18596210 PMC2544441

[66] Mehta D. Gunst S. J., “Actin polymerization stimulated by contractile activation regulates force development in canine tracheal smooth muscle,” J. Physiol. 519(3), 829–840 (1999). JPHYA7 0022-3751 10.1111/j.1469-7793.1999.0829n.x 10457094 PMC2269534

[67] Wegner A., “Head to tail polymerization of actin,” J. Mol. Biol. 108(1), 139–150 (1976). JMOBAK 0022-2836 10.1016/S0022-2836(76)80100-3 1003481

[68] Skruber K. Read T. A. Vitriol E. A., “Reconsidering an active role for G-actin in cytoskeletal regulation,” J. Cell Sci. 131(1), 203760 (2018). JNCSAI 0021-9533 10.1242/jcs.203760 PMC5818056 29321224

[69] Ohanian J. Pieri M. Ohanian V., “Non-receptor tyrosine kinases and the actin cytoskeleton in contractile vascular smooth muscle,” J. Physiol. 593(17), 3807–3814 (2015). JPHYA7 0022-3751 10.1113/jphysiol.2014.284174 25433074 PMC4575570

[70] Tang D. D., “Critical role of actin-associated proteins in smooth muscle contraction, cell proliferation, airway hyperresponsiveness and airway remodeling,” Respir. Res. 16, 134 (2015). 10.1186/s12931-015-0296-1 26517982 PMC4628321

[r71] Kutcher M. E. Herman I. M., “The pericyte: cellular regulator of microvascular blood flow,” Microvasc. Res. 77(3), 235–246 (2009). MIVRA6 0026-2862 10.1016/j.mvr.2009.01.007 19323975 PMC2668721

[72] Yamin R. Morgan K. G., “Deciphering actin cytoskeletal function in the contractile vascular smooth muscle cell,” J. Physiol. 590(17), 4145–4154 (2012). JPHYA7 0022-3751 10.1113/jphysiol.2012.232306 22687615 PMC3473273

[73] Boels P. J. Pfitzer G., “Relaxant effect of phalloidin on Triton-skinned microvascular and other smooth muscle preparations,” J. Muscle Res. Cell Motil. 13(1), 71–80 (1992). JMRMD3 0142-4319 10.1007/BF01738430 1313442

[74] Maekawa M. et al., “Signaling from Rho to the actin cytoskeleton through protein kinases ROCK and LIM-kinase,” Science 285(5429), 895–898 (1999). SCIEAS 0036-8075 10.1126/science.285.5429.895 10436159

[75] North A. J. et al., “Complementary distributions of vinculin and dystrophin define two distinct sarcolemma domains in smooth muscle,” J. Cell Biol. 120(5), 1159–1167 (1993). JCLBA3 0021-9525 10.1083/jcb.120.5.1159 8436588 PMC2119721

[76] Schor A. M. et al., “Pericytes derived from the retinal microvasculature undergo calcification in vitro,” J. Cell Sci. 97(3), 449–461 (1990). JNCSAI 0021-9533 10.1242/jcs.97.3.449 2074265

[77] Opazo Saez A. et al., “Tension development during contractile stimulation of smooth muscle requires recruitment of paxillin and vinculin to the membrane,” Am. J. Physiol. Cell Physiol. 286(2), C433–C447 (2004).1522-1563 10.1152/ajpcell.00030.2003 14576084

[78] Pavalko F. M. et al., “Phosphorylation of dense-plaque proteins talin and paxillin during tracheal smooth muscle contraction,” Am. J. Physiol. 268(3 Pt 1), C563–C571 (1995). AJPHAP 0002-9513 10.1152/ajpcell.1995.268.3.C563 7534979

[79] Kassianidou E. Kumar S., “A biomechanical perspective on stress fiber structure and function,” Biochim. Biophys. Acta 1853(11 Pt B), 3065–3074 (2015). BBACAQ 0006-3002 10.1016/j.bbamcr.2015.04.006 25896524 PMC4589434

[80] Herman I. M. D’Amore P. A., “Microvascular pericytes contain muscle and nonmuscle actins,” J. Cell Biol. 101(1), 43–52 (1985). JCLBA3 0021-9525 10.1083/jcb.101.1.43 3891763 PMC2113639

[81] Cipolla M. J. Gokina N. I. Osol G., “Pressure-induced actin polymerization in vascular smooth muscle as a mechanism underlying myogenic behavior,” FASEB J. 16(1), 72–76 (2002). FAJOEC 0892-6638 10.1096/cj.01-0104hyp 11772938

[82] Moreno-Dominguez A. et al., “Cytoskeletal reorganization evoked by Rho-associated kinase- and protein kinase C-catalyzed phosphorylation of cofilin and heat shock protein 27, respectively, contributes to myogenic constriction of rat cerebral arteries,” J. Biol. Chem. 289(30), 20939–20952 (2014). JBCHA3 0021-9258 10.1074/jbc.M114.553743 24914207 PMC4110300

[83] DeNofrio D. Hoock T. C. Herman I. M., “Functional sorting of actin isoforms in microvascular pericytes,” J. Cell Biol. 109(1), 191–202 (1989). JCLBA3 0021-9525 10.1083/jcb.109.1.191 2745546 PMC2115462

[84] Le Beux Y. J. Willemot J., “Actin- and myosin-like filaments in rat brain pericytes,” Anat. Rec. 190(4), 811–826 (1978). ANREAK 0003-276X 10.1002/ar.1091900404 345871

[85] Ho K. L., “Ultrastructure of cerebellar capillary hemangioblastoma. IV. Pericytes and their relationship to endothelial cells,” Acta Neuropathol. 67(3-4), 254–264 (1985). ANPTAL 1432-0533 10.1007/BF00687810 4050340

[86] Sawitzky H. et al., “The anti-proliferative agent jasplakinolide rearranges the actin cytoskeleton of plant cells,” Eur. J. Cell Biol. 78(6), 424–433 (1999). 10.1016/S0171-9335(99)80085-5 10430024

[87] Holzinger A. Blaas K., “Actin-dynamics in plant cells: the function of actin-perturbing substances: jasplakinolide, chondramides, phalloidin, cytochalasins, and latrunculins,” Methods Mol. Biol. 1365, 243–261 (2016). 10.1007/978-1-4939-3124-8_13 26498789 PMC4869834

[88] Coue M. et al., “Inhibition of actin polymerization by latrunculin A,” FEBS Lett. 213(2), 316–318 (1987). FEBLAL 0014-5793 10.1016/0014-5793(87)81513-2 3556584

[89] Brown S. S. Spudich J. A., “Mechanism of action of cytochalasin: evidence that it binds to actin filament ends,” J. Cell Biol. 88(3), 487–491 (1981). JCLBA3 0021-9525 10.1083/jcb.88.3.487 6894300 PMC2112756

[90] Flanagan M. D. Lin S., “Cytochalasins block actin filament elongation by binding to high affinity sites associated with F-actin,” J. Biol. Chem. 255(3), 835–838 (1980). JBCHA3 0021-9258 10.1016/S0021-9258(19)86105-7 7356663

[91] Odronitz F. Kollmar M., “Drawing the tree of eukaryotic life based on the analysis of 2,269 manually annotated myosins from 328 species,” Genome Biol. 8(9), R196 (2007). GNBLFW 1465-6906 10.1186/gb-2007-8-9-r196 17877792 PMC2375034

[92] Heissler S. M. Sellers J. R., “Various themes of myosin regulation,” J. Mol. Biol. 428(9 Pt B), 1927–1946 (2016). JMOBAK 0022-2836 10.1016/j.jmb.2016.01.022 26827725 PMC4860093

[93] Sellers J., “Myosins: a diverse superfamily,” Biochim. Biophys. Acta – Mol. Cell Res. 1496(1), 3–22 (2000). 10.1016/S0167-4889(00)00005-7 10722873

[94] Lee K. H. et al., “Interacting-heads motif has been conserved as a mechanism of myosin II inhibition since before the origin of animals,” Proc. Natl. Acad. Sci. U. S. A. 115(9), E1991–E2000 (2018). 10.1073/pnas.1715247115 29444861 PMC5834683

[95] Sweeney M. D. Ayyadurai S. Zlokovic B. V., “Pericytes of the neurovascular unit: key functions and signaling pathways,” Nat. Neurosci. 19(6), 771–783 (2016). NANEFN 1097-6256 10.1038/nn.4288 27227366 PMC5745011

[96] Kureli G. Erdener S. E. Dalkara T., “Organization of contractile proteins in pericytes suggest different flow regulatory functions,” in SfN Global Connectome; 11–13 Jan 2021; a Virtual Event (2021).

[97] Rauscher A. A. et al., “Targeting myosin by blebbistatin derivatives: optimization and pharmacological potential,” Trends Biochem. Sci. 43(9), 700–713 (2018). TBSCDB 0968-0004 10.1016/j.tibs.2018.06.006 30057142

[98] Somlyo A. P. Somlyo A. V., “Signal transduction by G-proteins, rho-kinase and protein phosphatase to smooth muscle and non-muscle myosin II,” J. Physiol. 522(2), 177–185 (2000). JPHYA7 0022-3751 10.1111/j.1469-7793.2000.t01-2-00177.x 10639096 PMC2269761

[99] Kimura K. et al., “Regulation of myosin phosphatase by Rho and Rho-associated kinase (Rho-kinase),” Science 273(5272), 245–248 (1996). SCIEAS 0036-8075 10.1126/science.273.5272.245 8662509

